# Supplementary material for: A Novel Interaction of Slug (SNAI2) and Nuclear Actin
Source: Cells. 2024 Apr 17;13(8):696. doi: 10.3390/cells13080696 (PMC11049500; doi:10.3390/cells13080696)
Supplement: Supplementary file 1 [file cells-13-00696-s001.zip › cells-2916739-supplementary.pdf]

Genes	Significant pairs	ANOVA p value	ANOVA q-value	log2 FC G vs.B	log2 FC E vs.B	log2 FC C vs.B	log2 FC G vs.C	log2 FC E vs.C	log2 FC G vs.E
ABCE1	B_G;C_G;E_G	0,000	0,011	-0,137	0,039	-0,004	-0,133	0,043	-0,177
ABHD10	B_G;C_G;E_G	0,000	0,014	-0,323	0,031	-0,003	-0,321	0,034	-0,354
ABTB2	B_G;E_G	0,003	0,041	-1,767	-0,111	-0,801	-0,966	0,690	-1,656
ADH5	B_G;C_G;E_G	0,000	0,009	-0,419	0,048	0,016	-0,435	0,032	-0,467
AHCY	B_G;C_G;E_G	0,001	0,016	-0,131	-0,007	-0,006	-0,125	-0,001	-0,124
AHSA1	B_G;C_G;E_G	0,000	0,008	-0,291	-0,043	-0,066	-0,225	0,023	-0,248
AKR1B1	B_G;C_G;E_G	0,002	0,035	-0,336	-0,045	-0,013	-0,323	-0,032	-0,291
AP2A1	B_G;C_G;E_G	0,004	0,050	-0,251	0,121	0,027	-0,278	0,094	-0,372
AP2B1	B_G;C_G;E_G	0,002	0,035	-0,225	0,073	0,019	-0,245	0,054	-0,298
ARF6	B_G;C_G;E_G	0,000	0,007	-0,292	0,065	0,039	-0,331	0,026	-0,356
ARFGEF2	B_G;C_G;E_G	0,002	0,031	-0,679	-0,175	-0,208	-0,471	0,033	-0,504
ARIH1	B_G;C_G	0,003	0,043	-0,390	-0,167	-0,132	-0,257	-0,035	-0,223
ARL6IP1	B_G;E_G	0,003	0,039	-0,341	0,175	-0,117	-0,224	0,292	-0,516
ARPC1A	B_G;C_G;E_G	0,000	0,013	-0,382	-0,038	-0,130	-0,251	0,092	-0,344
ARPC2	B_G;C_G;E_G	0,000	0,007	-0,462	-0,059	-0,104	-0,359	0,044	-0,403
ASF1A	B_G;C_G;E_G	0,002	0,029	-0,749	0,054	-0,069	-0,680	0,123	-0,803
ATL3	B_G;C_G;E_G	0,000	0,008	-0,321	0,149	0,065	-0,386	0,084	-0,470
ATP5F1C	B_G;C_G;E_G	0,004	0,050	-0,165	-0,007	-0,009	-0,156	0,002	-0,158
ATP5MK	B_G;C_G;E_G	0,001	0,020	-0,390	-0,059	-0,031	-0,359	-0,028	-0,331
ATP6V1C1	B_G;C_G;E_G	0,000	0,001	-0,381	0,032	0,163	-0,543	-0,131	-0,413
ATXN10	B_G;C_G;E_G	0,000	0,000	-0,338	0,080	-0,006	-0,332	0,086	-0,417
BANF1	B_G;C_G;E_G	0,002	0,031	-0,618	0,108	-0,067	-0,551	0,175	-0,726
BRD7	B_G;C_G;E_C;E_G	0,000	0,010	-0,558	0,081	-0,225	-0,333	0,306	-0,639
BUD23	B_G;C_G;E_G	0,003	0,035	-0,496	-0,140	-0,013	-0,483	-0,127	-0,356
BZW2	B_G;C_G;E_G	0,004	0,050	-0,295	-0,098	-0,056	-0,239	-0,042	-0,197
CAB39	B_G;C_G;E_G	0,004	0,046	-0,576	0,172	0,187	-0,762	-0,015	-0,748
CAND1	B_G;C_G;E_G	0,000	0,008	-0,196	0,084	0,085	-0,280	0,000	-0,280
CANX	B_G;C_G;E_G	0,000	0,009	-0,181	-0,010	0,011	-0,192	-0,021	-0,171
CCN1	B_G	0,002	0,035	-1,148	-0,580	-0,531	-0,617	-0,048	-0,568
CCT8	B_G;C_G;E_G	0,000	0,015	-0,136	0,000	-0,026	-0,110	0,026	-0,136
CD46	B_G;C_G;E_G	0,001	0,020	-1,183	0,135	-0,154	-1,029	0,289	-1,318
CD59	B_G;C_G;E_G	0,000	0,010	-0,723	0,320	0,028	-0,752	0,291	-1,043
CDC27	B_G;C_G;E_G	0,001	0,016	-0,331	0,070	-0,036	-0,295	0,106	-0,400
CDK1	G_B;G_C;G_E	0,003	0,039	0,099	-0,001	-0,058	0,157	0,057	0,100
CFL1	B_G;C_G;E_G	0,004	0,047	-0,441	0,091	0,014	-0,454	0,078	-0,532
CHORDC1	B_G;C_G;E_G	0,000	0,009	-0,329	0,022	0,044	-0,374	-0,022	-0,352
CHTOP	B_G;C_G;E_G	0,004	0,050	-0,494	0,123	0,022	-0,516	0,101	-0,617

CKAP2	B_G;C_G;E_G	0,000	0,015	-0,508	0,130	-0,041	-0,466	0,171	-0,638
CLTA	B_G;C_G;E_G	0,002	0,030	-0,688	0,059	-0,026	-0,663	0,085	-0,747
CLTC	B_G;C_G;E_G	0,000	0,008	-0,190	-0,049	-0,074	-0,115	0,025	-0,141
COL3A1	B_E;B_G;C_E;C_G	0,000	0,009	-0,601	-0,374	0,066	-0,667	-0,440	-0,227
COL4A1	B_G;C_G;E_G	0,001	0,026	-0,804	-0,064	-0,235	-0,568	0,172	-0,740
COPB1	B_G;C_G;E_G	0,000	0,007	-0,306	0,038	0,038	-0,344	0,000	-0,344
COPS6	B_G;C_G;E_G	0,000	0,000	-0,386	-0,034	0,001	-0,387	-0,035	-0,352
COX7C	B_G;C_G;E_G	0,000	0,006	-0,919	0,150	0,157	-1,076	-0,007	-1,069
CPOX	B_G;C_G;E_G	0,000	0,006	-0,910	0,139	0,015	-0,926	0,124	-1,050
CPT2	B_G;C_G;E_G	0,001	0,015	-0,462	0,019	-0,008	-0,453	0,028	-0,481
CSE1L	B_G;C_G;E_G	0,000	0,006	-0,216	0,058	0,020	-0,235	0,038	-0,274
CTTNBP2NL	G_B;G_C;G_E	0,000	0,014	0,204	-0,096	-0,066	0,270	-0,030	0,300
CUL1	B_C;B_E;B_G	0,001	0,022	-0,186	-0,176	-0,154	-0,032	-0,023	-0,009
CYCS	B_G;C_G;E_G	0,000	0,015	-0,386	0,067	0,116	-0,502	-0,049	-0,453
DAZAP1	B_G;C_G;E_G	0,000	0,014	-0,624	0,060	-0,088	-0,536	0,148	-0,684
DBI	B_G;C_G;E_G	0,001	0,015	-0,605	-0,090	-0,037	-0,568	-0,053	-0,515
DBN1	E_B;E_C;G_B;G_C	0,000	0,014	1,416	1,309	-0,065	1,482	1,374	0,107
DCUN1D1	B_G;C_G;E_G	0,000	0,012	-0,295	-0,004	-0,070	-0,225	0,066	-0,291
DDB1	B_G;C_G;E_G	0,000	0,000	-0,313	0,017	0,002	-0,315	0,015	-0,330
DDX39A	B_G;C_G;E_G	0,000	0,010	-0,455	0,097	-0,002	-0,453	0,099	-0,552
DDX39B	B_G;C_G;E_G	0,001	0,023	-0,433	0,157	-0,043	-0,389	0,201	-0,590
DDX3X	B_G;C_G;E_G	0,001	0,015	-0,404	-0,023	-0,059	-0,345	0,036	-0,381
DDX5	B_G;C_G;E_G	0,002	0,030	-0,192	0,063	0,007	-0,199	0,056	-0,255
DERA	B_G;C_G;E_G	0,001	0,016	-0,194	0,100	-0,034	-0,161	0,134	-0,294
DHCR24	B_G;C_G;E_G	0,000	0,015	-0,573	-0,018	0,055	-0,628	-0,073	-0,555
DHX29	B_G;C_G;E_G	0,000	0,013	-0,251	-0,093	-0,083	-0,168	-0,010	-0,158
DHX9	B_G;E_G	0,002	0,035	-0,377	0,130	-0,086	-0,290	0,217	-0,507
DIAPH1	B_E;B_G;C_G;E_G	0,000	0,000	-0,265	-0,101	-0,072	-0,193	-0,029	-0,164
DLST	B_G;C_G;E_G	0,000	0,002	-0,530	-0,054	-0,013	-0,518	-0,041	-0,477
DNAJA2	B_G;C_G;E_G	0,000	0,008	-0,436	0,054	0,039	-0,475	0,015	-0,490
DNAJC8	C_G;E_G	0,001	0,027	-0,129	0,093	0,092	-0,221	0,001	-0,222
DNAJC9	B_G;C_E;C_G	0,000	0,011	-0,210	-0,141	0,125	-0,335	-0,266	-0,069
DPM3	B_G;C_G;E_G	0,001	0,016	-0,890	0,132	0,012	-0,901	0,121	-1,022
DRAP1	B_G;C_G;E_G	0,002	0,031	-0,734	0,172	0,018	-0,752	0,154	-0,905
DYNC1H1	B_G;C_G;E_G	0,000	0,000	-0,145	-0,028	-0,028	-0,117	0,000	-0,118
EBP	B_G;C_G;E_G	0,000	0,014	-1,013	0,198	-0,059	-0,954	0,257	-1,211
EIF3E	B_G;C_G;E_G	0,002	0,035	-0,273	0,123	0,093	-0,366	0,030	-0,396
ELP2	B_G;C_G;E_G	0,001	0,019	-0,241	0,076	0,043	-0,284	0,034	-0,318

EPHX1	B_G;C_G;E_G	0,000	0,008	-0,638	0,180	0,183	-0,821	-0,003	-0,818
ERH	B_G;C_G;E_G	0,001	0,020	-0,643	0,214	-0,009	-0,635	0,222	-0,857
EWSR1	B_G;C_G;E_G	0,001	0,018	-0,679	0,163	0,060	-0,739	0,103	-0,842
EXOSC1	B_G;C_G;E_G	0,002	0,031	-0,438	0,048	-0,069	-0,370	0,116	-0,486
EXOSC10	B_G;C_G;E_G	0,000	0,007	-0,264	0,062	-0,059	-0,205	0,121	-0,326
EXOSC6	B_G;C_G;E_G	0,000	0,011	-0,305	0,100	-0,058	-0,247	0,159	-0,406
EXOSC7	B_G;C_G;E_G	0,002	0,035	-0,574	0,122	0,011	-0,584	0,112	-0,696
EXTL2	B_G;C_G;E_G	0,000	0,007	-0,526	-0,065	-0,086	-0,440	0,021	-0,461
FERMT2	B_G;C_G;E_G	0,000	0,007	-0,317	-0,022	0,000	-0,317	-0,023	-0,295
FGF2	B_G;E_C;E_G	0,000	0,015	-0,291	0,127	-0,089	-0,202	0,216	-0,418
FKBP1A	B_G;C_G;E_G	0,001	0,017	-0,845	0,043	0,056	-0,902	-0,013	-0,889
FKBP4	B_G;C_G;E_G	0,002	0,034	-0,283	-0,059	0,007	-0,290	-0,067	-0,223
FN3KRP	B_E;B_G;C_G	0,003	0,038	-0,475	-0,358	-0,125	-0,349	-0,232	-0,117
FOLR1	B_G;C_G;E_G	0,001	0,015	-0,515	0,331	0,178	-0,693	0,153	-0,847
FSTL1	B_G;C_G;E_G	0,000	0,014	-0,665	0,228	-0,190	-0,475	0,418	-0,893
GARS1	B_G;C_G;E_G	0,001	0,015	-0,140	-0,019	-0,038	-0,102	0,019	-0,120
GLG1	B_G;C_G;E_G	0,000	0,014	-0,544	0,084	0,107	-0,652	-0,023	-0,628
GLRX3	B_G;C_G;E_G	0,003	0,040	-0,277	0,019	0,043	-0,320	-0,024	-0,295
GLYR1	B_G;C_E;C_G	0,000	0,011	-0,292	-0,149	0,039	-0,331	-0,188	-0,143
GNG12	B_G;C_G;E_G	0,001	0,026	-0,295	0,189	0,125	-0,420	0,064	-0,483
GNPDA1	B_G;C_G;E_G	0,000	0,011	-0,518	-0,077	-0,151	-0,367	0,074	-0,441
GNPNAT1	E_B;E_C;E_G	0,002	0,029	-0,075	0,191	0,035	-0,110	0,156	-0,266
GOT1	B_G;C_G;E_G	0,000	0,000	-0,550	0,105	0,001	-0,551	0,104	-0,656
GPI	B_G;C_G;E_G	0,000	0,000	-0,344	0,044	0,013	-0,358	0,031	-0,389
GSPT1	B_G;C_G;E_G	0,000	0,013	-0,404	-0,037	-0,011	-0,394	-0,026	-0,368
GSTO1	B_G;C_G;E_G	0,001	0,027	-0,227	0,049	0,058	-0,285	-0,009	-0,276
GTF2A2	B_G;C_G;E_G	0,000	0,000	-3,458	0,220	-0,109	-3,349	0,329	-3,679
GTF2H1	B_G;C_G;E_G	0,003	0,041	-0,549	0,253	0,030	-0,579	0,223	-0,802
HEATR5B	B_C;B_E;B_G	0,001	0,015	-0,333	-0,535	-0,320	-0,013	-0,215	0,202
HEBP1	B_G;C_G;E_G	0,001	0,015	-0,273	0,018	0,007	-0,280	0,011	-0,291
HIBCH	B_G;C_G;E_G	0,002	0,028	-0,268	0,109	0,062	-0,330	0,048	-0,378
HLA-H	B_G;C_G;E_G	0,001	0,016	-0,703	0,382	0,304	-1,007	0,078	-1,085
HNRNPA0	B_G;E_G	0,003	0,044	-0,267	0,099	-0,056	-0,211	0,155	-0,366
HNRNPA1	B_G;C_G;E_G	0,000	0,006	-1,594	0,282	-0,102	-1,492	0,384	-1,876
HNRNPA3	B_G;C_G;E_G	0,001	0,021	-0,712	0,132	-0,152	-0,560	0,285	-0,845
HNRNPD	B_G;C_G;E_G	0,000	0,011	-0,249	0,058	-0,038	-0,211	0,096	-0,307
HNRNPU	B_G;C_G;E_G	0,002	0,028	-0,281	0,023	-0,085	-0,196	0,108	-0,303
HSD17B11	B_G;C_G;E_G	0,000	0,015	-0,569	-0,032	-0,042	-0,527	0,010	-0,537

HSD17B12	B_G;C_G;E_G	0,004	0,047	-0,323	-0,062	-0,019	-0,304	-0,043	-0,261
HSP90B1	B_G;C_G;E_G	0,004	0,049	-0,099	0,023	0,040	-0,139	-0,017	-0,122
HSPBP1	B_G;C_G;E_G	0,002	0,031	-0,204	0,061	0,004	-0,208	0,056	-0,265
HTRA1	B_G;C_E;C_G	0,001	0,015	-0,685	-0,347	0,081	-0,766	-0,428	-0,338
IARS1	B_G;C_G;E_G	0,000	0,007	-0,191	0,062	0,021	-0,212	0,041	-0,252
ID1	E_B;E_C;G_B;G_C	0,000	0,006	0,603	0,506	0,012	0,592	0,494	0,097
IDI1	B_G;C_G;E_G	0,000	0,011	-0,562	0,041	0,018	-0,579	0,024	-0,603
IPO5	B_G;C_G;E_G	0,002	0,029	-0,347	0,069	0,005	-0,352	0,063	-0,416
KPNA1	B_G;C_G;E_G	0,001	0,022	-0,457	-0,108	-0,160	-0,297	0,052	-0,349
KPNB1	B_G;C_G;E_G	0,000	0,012	-0,288	0,062	0,049	-0,338	0,012	-0,350
LARS1	B_G;C_G;E_G	0,000	0,005	-0,144	0,017	0,002	-0,146	0,015	-0,162
LAS1L	B_G;C_G;E_G	0,003	0,040	-0,211	0,071	-0,016	-0,195	0,088	-0,283
LDHA	B_G;C_G;E_G	0,000	0,011	-0,263	0,072	0,015	-0,279	0,057	-0,335
LIPA	B_G;E_G	0,003	0,040	-1,888	0,396	-0,951	-0,937	1,347	-2,284
LMBRD2	B_G;C_G;E_G	0,000	0,007	-1,146	0,004	-0,148	-0,998	0,152	-1,150
LSM8	B_G;C_G;E_G	0,002	0,033	-0,494	0,181	0,040	-0,534	0,141	-0,675
LUZP1	B_G;C_G;E_G	0,003	0,039	-0,276	0,093	0,010	-0,285	0,083	-0,368
M6PR	B_G;C_G;E_G	0,001	0,020	-0,494	-0,087	-0,033	-0,461	-0,054	-0,407
MAP4	B_G;C_G;E_G	0,000	0,011	-0,219	0,011	-0,014	-0,206	0,025	-0,230
MARCKS	B_G;C_G;E_G	0,001	0,026	-0,504	0,089	0,024	-0,528	0,065	-0,593
MDH1	B_G;C_G;E_G	0,003	0,038	-0,415	0,131	0,033	-0,448	0,098	-0,546
MED20	B_G;E_G	0,004	0,046	-0,381	-0,092	-0,160	-0,222	0,067	-0,289
MET	B_G;C_G	0,001	0,027	-0,264	-0,139	-0,091	-0,172	-0,048	-0,124
METAP2	B_G;C_G	0,001	0,015	-0,401	-0,201	-0,084	-0,317	-0,116	-0,200
MIX23	B_G;C_G;E_G	0,000	0,014	-0,308	0,097	0,056	-0,365	0,041	-0,405
MON2	B_G;C_G;E_G	0,002	0,030	-0,281	0,162	0,039	-0,321	0,123	-0,444
MRPL9	B_G;C_G;E_G	0,000	0,011	-0,319	0,037	0,045	-0,364	-0,008	-0,356
MSMO1	B_G;C_G;E_G	0,001	0,023	-1,714	0,209	0,126	-1,840	0,083	-1,923
MT-ATP6	B_G;C_G;E_G	0,000	0,011	-0,505	0,031	-0,063	-0,442	0,093	-0,535
MYBBP1A	B_G;C_G;E_G	0,000	0,014	-0,419	0,023	-0,051	-0,368	0,074	-0,442
MYO5A	E_B;E_C;G_B;G_C	0,002	0,034	2,373	2,282	0,516	1,857	1,766	0,091
NDUFB11	B_G;C_G;E_G	0,000	0,012	-3,369	0,308	0,036	-3,405	0,272	-3,677
NME1	B_G;C_G;E_G	0,001	0,020	-0,549	0,013	0,065	-0,613	-0,052	-0,561
NOMO1	B_G;C_G;E_G	0,000	0,008	-2,110	-0,093	-0,274	-1,836	0,180	-2,016
NOP53	B_G;E_G	0,001	0,025	-0,281	0,129	-0,089	-0,193	0,217	-0,410
NPC2	B_G;C_G;E_G	0,001	0,015	-0,464	0,064	0,109	-0,574	-0,045	-0,528
NRDC	B_G;C_G;E_G	0,000	0,014	-0,201	0,024	-0,016	-0,186	0,039	-0,225
NUP93	B_G;C_G;E_G	0,000	0,000	-0,159	-0,040	-0,017	-0,142	-0,023	-0,119

NXF1	B_G;C_G;E_G	0,000	0,007	-0,184	-0,027	0,034	-0,217	-0,061	-0,157
ODR4	B_G;E_G	0,004	0,048	-0,227	0,094	-0,111	-0,116	0,205	-0,321
PA2G4	B_G;C_G;E_G	0,000	0,007	-0,253	0,017	-0,015	-0,238	0,032	-0,270
PARVA	B_G;C_G;E_G	0,002	0,028	-0,545	0,135	-0,015	-0,530	0,150	-0,680
PBK	G_B;G_C;G_E	0,003	0,044	0,232	-0,071	0,036	0,196	-0,108	0,304
PCMT1	B_G;C_G;E_G	0,002	0,029	-0,423	0,090	0,022	-0,445	0,068	-0,513
PCYOX1	B_G;C_G;E_G	0,001	0,026	-0,302	0,001	0,065	-0,367	-0,064	-0,303
PICALM	B_G;C_G;E_G	0,001	0,015	-0,419	0,017	-0,049	-0,370	0,065	-0,435
PIGT	B_G;C_G;E_G	0,001	0,016	-0,265	0,055	0,038	-0,303	0,017	-0,320
PLK1	B_G;C_G;E_G	0,001	0,024	-0,497	0,008	-0,084	-0,413	0,092	-0,505
PLS3	B_G;C_G	0,003	0,041	-0,156	-0,046	0,014	-0,169	-0,060	-0,109
PLSCR3	C_B;C_E;G_B;G_E	0,001	0,019	2,340	-0,445	1,732	0,608	-2,177	2,785
POLR1B	B_C;B_G	0,004	0,050	-0,288	-0,123	-0,235	-0,053	0,113	-0,166
PPP1CA	B_G;C_G;E_G	0,000	0,007	-0,477	0,052	-0,020	-0,457	0,072	-0,529
PRC1	B_G;C_G;E_G	0,001	0,027	-0,542	-0,130	-0,129	-0,413	-0,001	-0,412
PRDX1	B_G;C_G;E_G	0,001	0,023	-0,354	0,087	0,005	-0,359	0,082	-0,441
PRKDC	B_G;C_G;E_G	0,001	0,015	-0,157	0,047	0,030	-0,187	0,017	-0,205
PRPS1	B_G;C_G;E_G	0,000	0,004	-0,482	0,111	-0,045	-0,437	0,156	-0,593
PRPS2	G_C;G_E	0,001	0,022	0,373	-0,286	-0,197	0,569	-0,090	0,659
PSMD1	B_G;C_G;E_G	0,002	0,035	-0,134	-0,021	-0,026	-0,108	0,005	-0,113
PSMD2	B_G;C_G;E_G	0,000	0,007	-0,287	0,015	-0,016	-0,271	0,031	-0,302
PSMD5	B_G;C_G;E_G	0,000	0,000	-0,299	0,042	-0,020	-0,280	0,062	-0,342
PSME1	B_G;C_G;E_G	0,001	0,015	-0,457	0,135	0,082	-0,539	0,053	-0,592
PTPA	B_G;C_G;E_G	0,001	0,018	-0,417	0,087	0,059	-0,476	0,028	-0,504
PYGL	B_E;B_G;C_E;C_G;E_	0,000	0,000	-0,205	-0,087	0,018	-0,223	-0,105	-0,119
RAB21	B_G;C_G;E_G	0,001	0,017	-0,337	0,008	0,000	-0,337	0,008	-0,345
RACK1	B_G;C_G;E_G	0,002	0,035	-0,414	0,010	-0,071	-0,343	0,081	-0,424
RAN	B_G;C_G;E_G	0,001	0,015	-0,269	0,094	0,007	-0,276	0,086	-0,363
RECQL	B_G;C_G;E_G	0,002	0,033	-0,524	0,020	-0,066	-0,458	0,086	-0,544
RFC5	B_G;C_G;E_G	0,000	0,015	-0,340	0,039	0,074	-0,414	-0,035	-0,379
RFXAP	C_B;C_G;E_B;E_G	0,002	0,033	-0,087	1,054	1,162	-1,249	-0,108	-1,141
RPA1	B_G;C_G;E_G	0,004	0,050	-0,579	0,025	-0,013	-0,566	0,038	-0,604
RPL26L1	B_G;C_G;E_G	0,000	0,010	-1,927	0,123	0,149	-2,076	-0,026	-2,050
RPL28	B_G;C_G;E_G	0,001	0,015	-0,738	0,197	0,103	-0,841	0,094	-0,936
RPL35A	B_G;C_G;E_G	0,003	0,040	-0,583	0,094	0,023	-0,607	0,070	-0,677
RPLP2	B_G;C_G;E_G	0,002	0,031	-0,287	0,074	0,003	-0,290	0,070	-0,361
RPN2	B_G;C_G;E_G	0,002	0,030	-0,206	-0,004	0,027	-0,232	-0,031	-0,201
RPS16	B_G;C_G;E_G	0,000	0,010	-0,239	0,045	0,036	-0,275	0,009	-0,284

RPS2	B_G;C_G;E_G	0,001	0,017	-0,182	-0,037	-0,052	-0,130	0,015	-0,145
RPS8	B_G;C_G;E_G	0,003	0,037	-0,269	-0,005	-0,023	-0,246	0,018	-0,264
RRAGA	B_G	0,002	0,028	-0,584	-0,272	-0,264	-0,320	-0,008	-0,312
RTN4	B_G;C_G;E_G	0,001	0,022	-0,504	-0,069	-0,016	-0,487	-0,053	-0,435
RTRAF	B_G;C_G;E_G	0,001	0,015	-0,369	0,109	0,029	-0,398	0,079	-0,477
S100A10	B_G;C_G;E_G	0,001	0,015	-0,694	-0,022	-0,012	-0,682	-0,011	-0,671
SAR1A	B_G;C_G;E_G	0,003	0,040	-0,269	0,092	-0,037	-0,231	0,129	-0,360
SAR1B	B_G;C_G;E_G	0,002	0,030	-0,212	0,028	0,052	-0,264	-0,024	-0,241
SEC23A	B_G;C_G;E_G	0,000	0,009	-0,207	-0,008	-0,038	-0,169	0,030	-0,200
SEC24C	B_G;C_G;E_G	0,001	0,020	-0,215	0,063	0,001	-0,215	0,062	-0,278
SEC61A1	B_G;C_G;E_G	0,002	0,035	-0,621	-0,036	0,017	-0,638	-0,053	-0,586
SEC61B	B_G;C_G;E_G	0,000	0,014	-1,828	0,131	-0,123	-1,706	0,253	-1,959
SENP3	B_E;C_E	0,001	0,016	-0,070	-0,172	0,014	-0,083	-0,186	0,102
SERPINE1	B_G;C_G;E_G	0,000	0,010	-0,895	-0,234	0,125	-1,020	-0,359	-0,661
SET	G_B;G_C;G_E	0,003	0,042	0,408	-0,003	0,009	0,399	-0,012	0,411
SIN3A	B_G;C_G;E_G	0,002	0,031	-0,187	-0,027	-0,008	-0,179	-0,019	-0,160
SKIC3	B_G;C_G;E_G	0,001	0,017	-0,160	0,017	0,037	-0,197	-0,020	-0,177
SLC25A11	B_G;C_G;E_G	0,001	0,015	-0,336	0,099	0,009	-0,345	0,090	-0,435
SLC25A5	B_G;C_G;E_G	0,001	0,015	-0,186	0,137	0,015	-0,200	0,122	-0,323
SLC35A4	B_G;C_G;E_G	0,000	0,014	-0,455	-0,007	0,052	-0,507	-0,059	-0,448
SLC38A2	G_B;G_C	0,004	0,050	0,466	0,292	0,123	0,343	0,169	0,173
SLC3A2	B_G;C_G;E_G	0,003	0,037	-0,208	-0,055	-0,057	-0,151	0,001	-0,153
SMU1	B_G;C_G;E_G	0,002	0,035	-0,396	0,108	-0,006	-0,390	0,114	-0,504
SPAG7	B_G;C_G;E_G	0,001	0,026	-0,293	0,120	0,061	-0,354	0,059	-0,414
SPCS3	B_G;C_G;E_G	0,001	0,020	-0,434	0,012	0,051	-0,485	-0,039	-0,446
SRP68	B_G;C_G;E_G	0,004	0,050	-0,182	-0,020	0,035	-0,217	-0,055	-0,162
STAG2	B_G;C_G;E_B;E_G	0,000	0,007	-0,162	0,123	0,060	-0,222	0,063	-0,285
STAU1	B_G;C_G;E_G	0,002	0,028	-0,384	-0,072	-0,037	-0,346	-0,035	-0,311
SYF2	B_G;C_G;E_G	0,000	0,011	-0,615	0,059	-0,026	-0,589	0,085	-0,675
TAB2	B_G;C_G;E_G	0,001	0,015	-1,071	0,086	-0,295	-0,776	0,381	-1,157
TARS1	B_G;C_G;E_G	0,001	0,016	-0,288	0,114	0,058	-0,347	0,055	-0,402
TBCB	G_B;G_C;G_E	0,004	0,045	0,364	-0,143	-0,022	0,385	-0,121	0,507
TFPI2	B_G;C_G;E_G	0,000	0,015	-1,934	-0,430	-0,089	-1,845	-0,342	-1,504
TIMM13	B_G;C_G;E_G	0,002	0,035	-0,145	0,084	0,050	-0,194	0,034	-0,228
TMA7	B_G;C_G;E_G	0,001	0,015	-2,827	0,172	0,309	-3,136	-0,137	-2,999
TMED2	B_G;C_G;E_G	0,002	0,029	-0,684	-0,082	0,051	-0,735	-0,133	-0,602
TMED3	B_G;C_G;E_G	0,001	0,015	-0,622	0,091	-0,107	-0,515	0,197	-0,713
TMEM43	B_G;C_G;E_G	0,002	0,035	-0,645	0,136	0,095	-0,741	0,041	-0,781

TMEM87A	E_B;E_G	0,001	0,026	-0,206	0,497	0,174	-0,380	0,323	-0,703
TMEM97	B_G;C_G;E_G	0,004	0,050	-0,692	0,079	0,108	-0,800	-0,029	-0,771
TNPO3	B_G;C_G;E_G	0,000	0,008	-0,474	0,116	0,148	-0,622	-0,032	-0,590
TPI1	B_G;C_G;E_G	0,000	0,010	-0,183	0,078	0,033	-0,216	0,045	-0,261
TRIM25	B_G;C_G;E_G	0,001	0,016	-0,326	0,038	-0,045	-0,281	0,082	-0,364
TSTD1	B_G;C_G;E_G	0,002	0,035	-0,933	0,146	0,293	-1,226	-0,146	-1,080
TXN	B_G;C_G;E_G	0,001	0,025	-0,805	0,116	-0,042	-0,763	0,158	-0,921
TXNDC5	B_G;C_G;E_G	0,000	0,014	-0,451	0,075	0,008	-0,459	0,067	-0,526
UCHL5	C_G;E_G	0,002	0,034	-0,106	0,113	0,049	-0,155	0,065	-0,220
VCL	B_G;C_G;E_G	0,002	0,035	-0,609	0,133	0,044	-0,653	0,089	-0,742
VDAC3	B_G;C_G;E_G	0,003	0,040	-0,254	0,003	0,009	-0,264	-0,007	-0,257
WDFY1	B_G;C_G;E_G	0,001	0,015	-0,186	0,070	0,033	-0,219	0,038	-0,256
WDR5	B_G;C_G;E_G	0,003	0,044	-0,345	0,026	0,147	-0,492	-0,121	-0,371
WDR6	B_G;C_G;E_G	0,000	0,011	-0,425	0,078	-0,052	-0,373	0,129	-0,502
XPO1	B_G;C_G;E_G	0,000	0,009	-0,219	0,059	-0,002	-0,217	0,060	-0,278
YARS1	B_G;C_G;E_G	0,003	0,041	-0,115	-0,017	-0,008	-0,106	-0,009	-0,097
YWHAE	B_G;C_G;E_G	0,003	0,043	-0,212	0,051	0,014	-0,226	0,036	-0,263
ZC3H18	B_G;C_G;E_G	0,002	0,034	-0,327	-0,007	-0,041	-0,285	0,035	-0,320

Supplementary Table S1: Multiple comparisons of different experimental conditions with respect to proteins interacting with actin. Only proteins showing significant changes between at least two groups are listed. B: Untreated control, C: Treatment with latB, D: UV irradiation, no repair, E: UV irradiation, no repair + LatB treatment, G: UV irradiation, 1h repair and LatB treatment. Comparisons with the other groups (e.g. F: UV treatment plus 1h repair) elicited no significant changes.
